# Toward Standardization of Electrophysiology and Computational Tissue Strain in Rodent Intracortical Microelectrode Models

**DOI:** 10.3389/fbioe.2020.00416

**Published:** 2020-05-08

**Authors:** Shreya Mahajan, John K. Hermann, Hillary W. Bedell, Jonah A. Sharkins, Lei Chen, Keying Chen, Seth M. Meade, Cara S. Smith, Jacob Rayyan, He Feng, Youjoung Kim, Matthew A. Schiefer, Dawn M. Taylor, Jeffrey R. Capadona, Evon S. Ereifej

**Affiliations:** ^1^Department of Electrical Engineering and Computer Science, University of Michigan, Ann Arbor, MI, United States; ^2^Department of Biomedical Engineering, Case Western Reserve University, Cleveland, OH, United States; ^3^Advanced Platform Technology Center, Louis Stokes Cleveland Veterans Affairs Medical Center, Cleveland, OH, United States; ^4^Veteran Affairs Ann Arbor Healthcare System, Ann Arbor, MI, United States; ^5^Department of Biomedical Engineering, University of Michigan, Ann Arbor, MI, United States; ^6^Department of Mechanical Engineering, University of Michigan, Ann Arbor, MI, United States; ^7^Department of Neuroscience, The Cleveland Clinic, Cleveland, OH, United States; ^8^Department of Neurology, University of Michigan, Ann Arbor, MI, United States; ^9^Functional Electrical Stimulation Center, Louis Stokes Cleveland Veterans Affairs Medical Center, Cleveland, OH, United States

**Keywords:** rodent model, intracortical microelectrodes, electrophysiology, tissue strain, brain, finite element model

## Abstract

Progress has been made in the field of neural interfacing using both mouse and rat models, yet standardization of these models’ interchangeability has yet to be established. The mouse model allows for transgenic, optogenetic, and advanced imaging modalities which can be used to examine the biological impact and failure mechanisms associated with the neural implant itself. The ability to directly compare electrophysiological data between mouse and rat models is crucial for the development and assessment of neural interfaces. The most obvious difference in the two rodent models is size, which raises concern for the role of device-induced tissue strain. Strain exerted on brain tissue by implanted microelectrode arrays is hypothesized to affect long-term recording performance. Therefore, understanding any potential differences in tissue strain caused by differences in the implant to tissue size ratio is crucial for validating the interchangeability of rat and mouse models. Hence, this study is aimed at investigating the electrophysiological variances and predictive device-induced tissue strain. Rat and mouse electrophysiological recordings were collected from implanted animals for eight weeks. A finite element model was utilized to assess the tissue strain from implanted intracortical microelectrodes, taking into account the differences in the depth within the cortex, implantation depth, and electrode geometry between the two models. The rat model demonstrated a larger percentage of channels recording single unit activity and number of units recorded per channel at acute but not chronic time points, relative to the mouse model Additionally, the finite element models also revealed no predictive differences in tissue strain between the two rodent models. Collectively our results show that these two models are comparable after taking into consideration some recommendations to maintain uniform conditions for future studies where direct comparisons of electrophysiological and tissue strain data between the two animal models will be required.

## Introduction

Intracortical microelectrodes (IMEs) allow for direct interfacing with neuronal populations, thereby enabling the exploration of neuronal function, neurological diseases, and potential therapies (Wise and Angell, 1975; Schwartz, 2004; Kipke et al., 2008). Intracortical microelectrodes are able to record and transmit electrical impulses directly from neurons in the brain (Renshaw et al., 1940). Recorded electrical impulses can then be used in numerous applications, including being translated into control signals for prosthetic devices to restore function (Hochberg et al., 2006; Schwartz et al., 2006; Gilja et al., 2015). When used in brain–machine interfacing (BMI) applications, IMEs have enabled quadriplegic and amyotrophic lateral sclerosis (ALS) patients to control external neuroprosthetic devices as well as neuromuscular stimulation systems that restore movement to their own limbs (Gilja et al., 2015; Schroeder and Chestek, 2016; Ajiboye et al., 2017). Intracortical microelectrodes have also been an essential part of many basic neuroscience studies and have significantly advanced our understanding of natural brain function.

The usefulness of IMEs depends on the ability to reliably record the electrical signals from many individual neurons over time (Wise, 2005; Kozai et al., 2015a; Ajiboye et al., 2017). The ability of IMEs to detect isolatable action potentials from individual neurons is directly dependent on the distance between healthy neuronal cell bodies and the microelectrode recording site (Buzsáki, 2004). The inflammatory response to implanted electrodes is thought to limit the ability to record consistent and reliable signals over time and contribute to electrode failure (Biran et al., 2005; McConnell et al., 2009; Potter et al., 2012; Kozai et al., 2015b). The complex inflammatory response occurring after electrode implantation results in decreased recording quality within weeks, and a continued decline often leading to complete loss of detectable action potentials within a few years (Chestek et al., 2011; Jorfi et al., 2015; Kozai et al., 2015b).

The initial insertion of IME causes breaching of the blood–brain barrier (BBB), which leads to injury of the local brain parenchyma (Potter et al., 2013; Saxena et al., 2013). Damage to the BBB is believed to be chronically agitated as stiff implants move against the natural micromotions of the brain observed during respiration and activity-dependent vasculature changes (Gilletti and Muthuswamy, 2006; Harris et al., 2011; Nguyen et al., 2014; Zhang et al., 2014; Prodanov and Delbeke, 2016). The breached BBB allows for the infiltration of blood-borne cells and the initiation of an inflammatory response (Ravikumar et al., 2014b; Bedell et al., 2018a). Chronic implantation of the IME results in a frustrated phagocytic event, causing a build-up of pro-inflammatory factors (He et al., 2007; Skousen et al., 2015). Accumulations of pro-inflammatory molecules causes oxidative damage to the surrounding cells, tissue, and the IME (Ereifej et al., 2018; Bennett et al., 2019). Within weeks, the inflammatory response results in a glial scar which creates a barrier believed to reduce the ability of the IME to consistently record signals from local neurons (McConnell et al., 2009; Ravikumar et al., 2014b; Kozai et al., 2015b; Nguyen et al., 2016).

The early failures of IMEs have sparked substantial research in understanding electrode failure by utilizing several animal models and novel techniques. Studies involving feline models have shown unreliable and inconsistent electrode performance, with variability in recordings for up to 2 months, due to the instability of the electrode–tissue interface (Rousche and Normann, 1998; Xindong et al., 1999, 2006). Utilization of primates in IME studies have shown the potential clinical applications of the electrodes, but unfortunately suffer from the same decline in recording performance (Chestek et al., 2011; Barrese et al., 2013, 2016). Recent work introducing a marmoset model also displayed similar signal degradation found in traditional animal models (Debnath et al., 2018). Rodent models yield similar electrode reliability results compared to the larger animal alternatives. However, the smaller sized animals offer a less sentinel species with lower costs and smaller housing requirements, enabling larger sample sizes for increased statistical power. Therefore, rodents continue to be the most commonly utilized animal model to investigate the performance and integration of IME.

In turn, rats have been employed for years to better understand the failure mechanism after IME implantation (Vetter et al., 2004; Zhong and Bellamkonda, 2007; McConnell et al., 2009; Winslow and Tresco, 2010; Potter et al., 2012; Prasad et al., 2014; Nolta et al., 2015; Bennett et al., 2018; Ereifej et al., 2018; Salatino et al., 2019). Moreover, recent work has shown the ability to use rats to investigate motor functions following implantation of neural probes in the motor cortex (Goss-Varley et al., 2017, 2018). It is thought that rats are utilized more frequently in experiments compared to mice, due to their stronger physiological similarities to humans (Shanks et al., 2009) and ease in surgical procedures due to larger size (Barre-Sinoussi and Montagutelli, 2015).

While rat models have proven useful, there has been a recent move toward the use of mice when studying the acute and chronic performance of developing IME technologies. For examples, transgenic knock out studies are commonly performed in mice over rats due to cost and the availability of “off-the-shelf” engineered animals. Transgenic animal models enable researchers to target and better understand specific genes that may affect electrode performance (Kozai et al., 2014; Bedell et al., 2018a, b; Hermann et al., 2018a, b). Additionally, mouse models have shown promise with optogenetic studies, aiding the ability to correlate the electrophysiology a specific cell to the observed behaviors (Anikeeva et al., 2011; Pashaie et al., 2014; Park et al., 2017). Mice are also ideal candidates for 2-photon microscopy, due to the smaller size of their brain allowing for live imaging into deeper structures of the brain. For example, 2-photon microscopy with mice has been used in the field of neural engineering to investigate the live cellular response along the depth of the implanted IME (Kozai et al., 2012, 2016).

As can be seen from the aforementioned examples, there are advantages to using both rats and mice when studying IME performance and the evoked tissue response. In fact, our lab has used both interchangeably (Ereifej et al., 2017, 2018; Bedell et al., 2018a, 2019; Hermann et al., 2018a; Mahajan et al., 2019). The initial intent of this study was to perform an internal check of consistency to ensure that we were justified in using both rodent models. Here, we report on our “internal audit” as a way to help standardize the field and ease any similar concerns, while preventing the unnecessary duplication of animal research. It is critical to validate the scaling and translation of findings from the mouse model to inform the design of experiments using rat models, and vice versa. Of note, other fields of research such as, diabetes, orthopedic, gene sequencing, among others, have shown the importance of validating the ability to use different animal models and compare them to one another while relating to the clinical models (Working, 1988; Giannobile et al., 1994; Newman et al., 1995; Yan et al., 2011; Islam et al., 2013). Particularly, previous work from our lab attempting to account for the animal model interchangeability has shown negligible differences of histological outcomes for glial cell activation, neuronal density and BBB breaching between the two models at initial (2 weeks) and chronic (16 weeks) time points (Potter-Baker et al., 2014). However, we have yet to investigate whether there are differences observed in electrophysiological recordings between the rat and mouse model. It is also of great interest to evaluate any implications the implanted electrodes have on the mechanical strain exerted on the adjacent brain tissue. It is hypothesized that there may be a direct relationship between the size of the electrode relative to the brain tissue and the resultant strain on the brain tissue. Therefore, this study is the first to utilize finite element modeling to compare tissue strain caused by IMEs in both the rat and mouse model, as well as comparisons of the electrophysiological recordings made from the two models over 8 weeks under similar conditions.

## Materials and Methods

### Intracortical Microelectrode Implantation Procedure

Similar silicon, single shank, 16 channel IMEs from NeuroNexus were utilized in both rodent models but with different inter-electrode-contact spacing to account for differences in cortical thickness. Specifically, the rat model used probe model # A1 × 16-3mm-100-177-Z16, with inter-contact spacing of 100 μm, while the mice were implanted with probe model # A1 × 16-3mm-50-177-Z16, which had an inter-contact spacing of 50 μm. Prior to implantation, electrode impedance magnitudes were measured at 1 kHz in saline and verified to the manufacturer’s values. Electrodes were then washed with a mixture of 95% ethanol and deionized water for 5 min and air dried. Implants were then sterilized using the standard ethylene oxide (EO) gas protocol: 54.4^*o*^ F, 1 h sterile time and 12 h aerate (Ravikumar et al., 2014a).

The Institutional Animal Care and Use Committees (IACUC) at the Louis Stokes Cleveland Veterans Affairs Medical Center (LSCDVAMC) and Case Western Reserve University (CWRU) approved all animal procedures. All rat work was performed at LSCDVAMC by one surgeon, while mouse work was performed at CWRU by two other surgeons; all three surgeons have the same expert level competency for performing these surgeries. Six male Sprague Dawley rats (8–10 weeks old and weighing ∼225 g) and 14 male/female C57/BL mice (8–10 weeks old and weighing ∼20g) were utilized in this study. Implantation procedures were similar for both animal models with a few minor differences due to protocol requirements for other ongoing studies. Both animal models used isoflurane to anesthetize the animals to the surgical plane prior to surgery, (3.5% in 1.5 L/min O_2_ for rats and 3% in 1.0 L/min O_2_ for mice). Surgical procedures for mice were performed in a class II sterile hood using microisolator techniques, whereas the rat surgeries were done on an open surgical table using aseptic techniques. Animals were prepped for surgery by first shaving the implantation site, and then sterilizing the area with alternating wipes of betandine for mice and chlorhexidine gluconate (CHG) for rats, and isopropanol. Marcaine (0.2 mL of 0.25% Marcaine) was administered subcutaneously (SQ) around the surgical site as a topical anesthetic. For antibiotics and analgesic, Cefazolin (25 mg/kg in rats), Carprofen (5 mg/kg for rats), and Meloxicam (2 mg/kg in mice) were administered subcutaneously. Animals were then mounted on a stereotaxic frame and maintained at the surgical plane by inhalation of 0.5–2% isoflurane through a nosecone throughout the surgery.

In both animal models, an incision was made down the midline of the scalp and the skin was retracted to expose the skull. The skull was cleaned of the periosteum, dehydrated using hydrogen peroxide, and primed using Vetbond animal tissue adhesive. Three craniotomies were drilled, one each for the ground wire, reference wire and electrode. A 0.45 mm drill bit was used for all craniotomies, except for the electrode craniotomy in rats, which was 2 mm in diameter. Because the dura was too thick for the silicon electrodes to penetrate in the rat model, the dura was reflected prior to implanting the electrode in the rat. Due to the size difference between the rat and mouse skull, the coordinates for the craniotomies differed slightly. In rats, the ground wire was 1.5 mm lateral to midline and 1.5 mm posterior to bregma, the reference wire was 1.5 mm lateral to midline and 5.5 mm posterior to bregma, and the electrode craniotomy was on the contralateral side, 2 mm lateral to midline and 2 mm anterior to bregma (forelimb primary motor cortex). In mice, the ground wire was 1.5 mm lateral to midline and 1 mm rostral to the bregma and the reference wire was 1.5 mm lateral to midline and 1 mm caudal to the bregma, and the electrode craniotomy was on the contralateral side, 1.5 mm lateral and 0.5 mm anterior or posterior to the bregma (motor/sensorimotor cortex). The ground and reference wires were manually inserted and secured to the skull (rats used Teets cold cure dental cement and mice used silicone elastomer and Stoelting dental cement). The electrodes were implanted using a Kopf micromanipulator. In rats, the implant insertion rate was 100 μm (the distance between channels) every 1–2 min up to an approximate depth of 2000 μm, which ensured that the length of the electrode containing the 16 contacts spanned across layers III–V of the cortex containing the majority of the detectable pyramidal cells. In mice, the electrodes were inserted in increments of 50 μm (the distance between channels) at a rate of 10 μm/s, with intervals of 10–30 s in between. The electrode was implanted to a depth of approximately 1000 μm, to ensure that the contact sites of the electrode were present in cortical layers III–V. The electrode craniotomies were then sealed with a silicone elastomer (Kwik-Sil, World Precision Instruments) and a dental acrylic (Teets in rats, Stoelting in mice) was used to secure the electrode connector and form a sturdy headcap. Post-operative care and monitoring was provided the week following surgery, with administration of analgesia and antibiotics for up to three days following surgery.

### Electrophysiological Recordings

Awake and freely moving tethered electrophysiological recordings were collected from the animals after surgical recovery. Recordings were taken using the TDT RZ5D BioAmp Processor recording system for the rat study, and TDT RX5 Pentusa Processor for the mouse studies. Prior to recordings, animals were anesthetized using 3% isoflurane when connecting the headstage cable to minimize head movement that could lead to connector damage. Ten minute recordings from each rat were collected twice per week starting the day after surgery continuing for 8 weeks, whereas 3 min recordings were obtained from each mouse twice per week beginning 5 days post-surgery until 16 weeks post-implantation. For direct comparison to the rat model, only the same 8 week duration is presented here.

### Signal Processing

The electrophysiological data were processed as previously published, by implementing a sampling rate of 24.4 kHz, a bandpass filter spanning 300 Hz to 3 kHz and a common average reference algorithm (Bedell et al., 2018a; Hermann et al., 2018b). In order to account for some inherent variance in the final implanted electrode depth between animals (e.g., due to differences in brain swelling during implantation), performance metrics were calculated using the most active electrode contacts presumed to span the layers with the large pyramidal neurons. In rats the 10 consecutive channels having the most spiking activity (defined by the highest sum of average units over the 8 week study) were utilized for the final rat data assessment. Due to the smaller cortical thickness in mice, metrics for the mice were calculated using the eight consecutive channels with the highest sum of average units over time.

To address artifacts attributed to motion, moisture shorting head stage contacts, and inconsistent connections between recording equipment and arrays in an objective manner, blinded artifact removal techniques were applied as previously described in detail (Bedell et al., 2018a; Hermann et al., 2018b). Artifact removal techniques administered by reviewer without knowledge of type of subject or time point included removal of time segments, channels, or recording sessions. In both rats and mice, the removed days and channels were randomly distributed across channels, days and animals groups (rat data removed = 1.04% of recording days and 1.25% of individual channels; mouse data removed = 9.7% of recording days and 2.6% of individual channels).

Following artifact removal, spikes were detected with a threshold of 3.5 standard deviations from the mean voltage. Snippets of data spanning 12 samples pre-threshold crossing to 24 samples post-threshold crossing were clustered by the unsupervised sorting algorithm, Wave_clus (Quiroga et al., 2004; Bedell et al., 2018a; Hermann et al., 2018b). The number of single unit clusters identified by the sorting algorithm was logged for each channel of the microelectrode array. Information about the number of clusters detected and signal quality of each cluster was used to generate five metrics for quantitative comparison (as described in Bedell et al. (2018a), Hermann et al. (2018b). The first three metrics were calculated from all “working” channels (i.e., channels having impedances and noise levels consistent with intact connections): (1) percentage of working channels detecting single units, (2) average number of units per working channel, and (3) background noise level averaged across all working channels where noise level was calculated as twice the standard deviation of the background activity after time windows containing spikes and artifacts were removed [robust methods from Quiroga et al. (2004)]. Two additional metrics were calculated from the subset of channels detecting isolatable single units: (4) peak-to-peak maximum amplitude of each unit’s mean waveform averaged across all units on a given channel and (5) signal-to-noise ratio (SNR) averaged across units where the “signal” is each units mean peak-to-peak amplitude and noise is calculated as in (3).

### Strain Model

**FIGURE 1:**
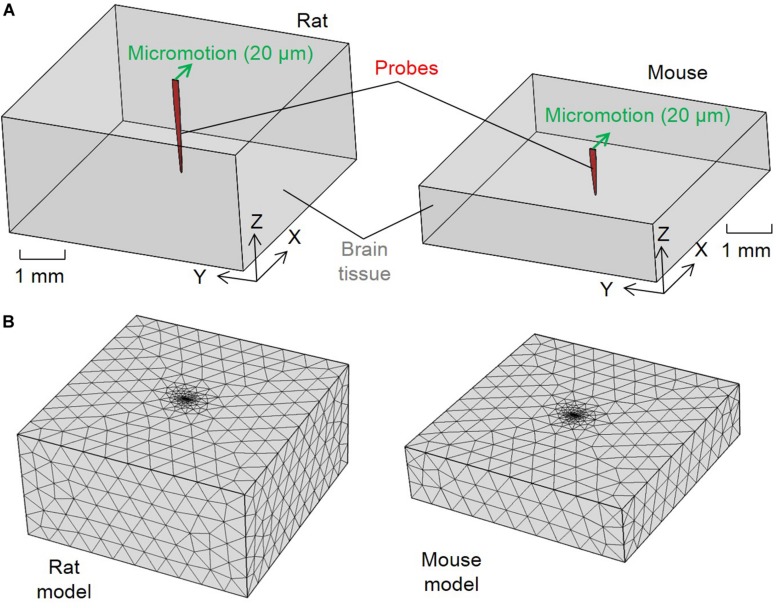
Configurations for the finite element method strain models. **(A)** Schematics of the model configurations and set up of the micromotion and **(B)** tetrahedron mesh of the rat and mouse models.

To investigate the difference between rats and mice in the tissue strain caused by brain micromotion after electrode implantation, three-dimensional (3D) finite-element models simulating interfacial strains induced by brain micromotion from respiration and vasculature were developed. We utilized a model that consisted of two components: a silicon-based single probe shank (dimensions and insertion depths relative to the electrodes used in the rat and mouse surgeries, respectively) and surrounding brain tissue (dimensions relative to animal’s respective cortex size), as shown in Figure 1. The cross-sectional dimensions of the brain tissue surrounding the electrode are two magnitudes higher than the probe cross-sectional area, ensuring that the boundaries will have negligible impact on the results, and only the depth of the electrode in relation to the cortex will be a variable taken into consideration in this model. In this study, the cortex was assumed to be linearly elastic with isotropic material properties to induce the primary strains in the surrounding tissue after electrode implantation. Table 1 summarizes the dimensions and material properties used for the two components in both models, as described previously (Subbaroyan et al., 2005; Nguyen et al., 2014). Recent work investigating the mechanical properties of various brain tissue specimens utilized in traumatic brain injury models, revealed no significant differences of viscoelastic properties between the rat and mouse cortical tissue (MacManus et al., 2017). Thus suggesting it is suitable to use the same Young’s modulus and Poisson’s ratio for the rat and mouse in our computational model.

**TABLE 1:** Parameters used in the finite-element models to simulate micromotion and strain from implanted silicon probe shank in rat and mouse brain.

Components	Parameter	Rat model		Mouse model
Brain Tissue	Young’s Modulus		6 KPa	
	Poisson’s ratio		0.43	
	Dimension (X × Y × Z)	5000 μm × 5000 μm × 2500 μm		5000 μm × 5000 μm × 1250 μm
Silicon Probe Shank	Young’s Modulus		200 GPa	
	Poisson’s ratio		0.27	
	Dimension	Thickness = 15 μm, Max width = 123 μm narrowing down to 33 μm over a 1500 μm length and a 50 μm long tapering end		Thickness = 15 μm, Max width = 123 μm narrowing down to 33 μm over a 750 μm length and a 50 μm long tapering end
	Inter-channel length	100 μm		50 μm
	Insertion depth	2000 μm		1000 μm
	Prescribed displacement (due to micromotion)	20 μm		

The brain and electrode components were treated as one connected unit in both models by assuming tied contacts at the probe–brain interface. The bottom and side outer surfaces of the brain tissue were fixed in all directions as model boundary conditions. To simulate the relative tangential tethering, micromotion between the skull and brain tissue from an implant fixed to the skull, a 20 μm displacement along the positive X direction was applied to the top of the probe’s shank. This simulated the displacement due to the relative micromotion between the skull and brain tissue, as marked by the green arrows in Figure 1A. Similar models investigating tissue strain after microelectrode implantation utilized 20 μm micromotion, which falls in the range of vascular (2–4 μm) and respiratory (10–30 μm) micromotion, as well as the calculated induced electrode displacement (20 μm) following rotational acceleration of an animal’s head (Muthuswamy et al., 2003; Lee et al., 2005; Subbaroyan et al., 2005; Gilletti and Muthuswamy, 2006; Sridharan et al., 2013; Nguyen et al., 2014).

The two components were meshed into tetrahedron elements (Figure 1B). Brain tissue elements away from the interface for both the rat and mouse models had mesh sizes of about 500 μm. A finer mesh was used to model the probe and area surrounding the interface elements with an average size of 83.27μm. The meshing methodology generated in total 2,589 nodes and 13,495 elements for the rat model and 1,507 nodes and 7,347 elements for the mouse model. Strain distribution induced in the meshed models are as shown in Figure 1B. Strain distributions induced by the micromotion were solved using COMSOL Multiphysics software (COMSOL, Burlington, MA, United States). Strain distribution was described by von Mises strain, ε_*e*_, which was calculated following the equation (Lee et al., 2005):

(1) $$\varepsilon_{e}=\frac{1}{1+\nu }{\left\{\frac{1}{2}\left[{\left(\varepsilon_{1}-\varepsilon_{2}\right)}^{2}+{\left(\varepsilon_{2}-\varepsilon_{3}\right)}^{2}+{\left(\varepsilon_{3}-\varepsilon_{1}\right)}^{2}\right]\right\}}^{\frac{1}{2}}$$

where ν is the Poisson’s ratio of the brain tissue andε_1_, ε_2_, and ε_3_ are first, second, and third principal strains correspondingly from output of the finite element method model as described in section “Strain Model”. The von Mises strain fields obtained through Eq. (1) were then normalized by dividing all of the von Mises strain values by the maximum induced von Mises strain values across the entire models for both rat and mouse. The maximum induced von Mises strain value was found at the probe tip of the mouse model. The final von Mises strain values then ranged between zero and one allowing for impartial comparison between the two models.

### Statistical Analysis

For statistical analysis of the five electrophysiology metrics, a general linear model in Minitab 16 (Minitab Inc., State College, PA, United States) was utilized to allow for comparisons between conditions (time and animal group) as fixed factors. Time was grouped in three ranges, total time (weeks 1–8), acute (weeks 1–2), and chronic (weeks 3–8) to represent the phases of the neuroinflammatory response at the neural interface (Reichert, 2007; Potter et al., 2012). To account for repeated measures, each individual rat or mouse was nested within its respective animal group. The terms of the model were the animal group and the different time intervals. Significance was defined as *p* < 0.05. Statistical analysis on the finite element strain models was performed by a Pearson correlation between the rat and mouse model.

## Results

### Electrophysiological Recordings

#### Variability in Recordings

**FIGURE 2:**
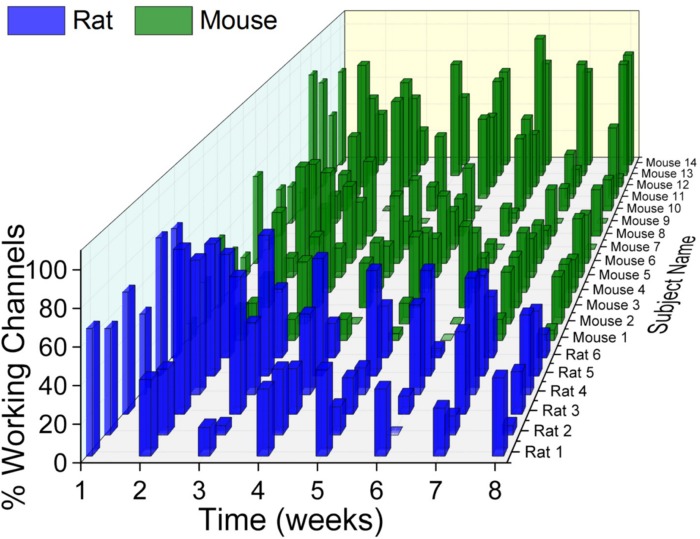
Electrophysiological recordings variability. There was substantial variability within and across animal models as well as across time. With few exceptions, there was a universal trend for recording quality to decline over time.

One of the main challenges to successful IME implementation is obtaining consistent recording quality. Inconsistent recording quality over time within the same animal, as well as between animals within the same group has been observed with previous studies utilizing one animal species (Rousche and Normann, 1998; Xindong et al., 1999, 2006; Chestek et al., 2011; Barrese et al., 2013, 2016; Michelson and Kozai, 2018). To our knowledge, a direct comparison of electrophysiological recording quality between two different animal species has not been previously reported. Here, our evaluation of the rat and mouse models revealed that both have variability within their own animal group, over time, and compared to one another (Figure 2).

#### Electrophysiological Results

Electrophysiological recordings from the rat model were compared to the mouse model over the entire course of time (weeks 1–8), as well as group based on the time course of inflammatory events following device implantation: acute (weeks 1–2) and chronic (weeks 3–8) time points.

**FIGURE 3:**
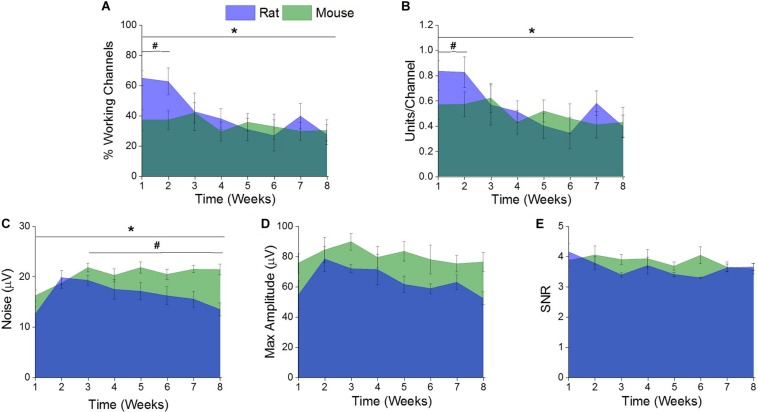
Comparison of electrophysiological recording metrics between rat and mouse models. There were significant differences between the **(A)** % working channels and **(B)** units per channel between the rat and mouse recordings over the entire eight weeks. (* indicates a *p* < 0.05). Specifically, there were significantly more **(A)** % working channels and **(B)** units per channel recorded from the rat recordings compared to the mouse recordings at acute time points (1–2 weeks) (# indicates a *p* < 0.05). There were significant differences in **(C)** noise between the rat and mouse recordings over the entire eight weeks. (*indicates a *p* < 0.05). Specifically, there was significantly more noise from the mouse recordings compared to the rat between the acute (1–2 weeks) and chronic (3–8 weeks). (#indicates a *p* < 0.05). There were no significant differences between the recorded **(D)** maximum amplitude and **(E)** signal-to noise ratios (SNR) between the rat and mouse recordings over the entire eight weeks or when compared at acute versus chronic time intervals.

There was a significant difference (^∗^indicates *p* < 0.05) in the percentage of channels recording single units and the single units recorded per channel between the rat and mouse models over the entire 8 weeks (Figures 3A,B). Further evaluation revealed there were significantly higher (#indicates *p* < 0.05) percentage of channels recording single units and single units recorded per channel in the rat model during the acute time point compared to the mouse model (Figures 3A,B). There were no significant differences of percentage of channels recording single units or single units recorded per channel between the two rodent models during the chronic time points (Figures 3A,B).

There was a significant difference (^∗^indicates *p* < 0.05) of recorded noise between the rat and mouse models over the entire 8 weeks (Figure 3C). Yet, there were no significant differences in recorded noise between the two rodent models during the acute time points (Figure 3C). It was found that there was significantly more (#indicates *p* < 0.05) noise recorded in the mouse model compared to the rat model during the chronic time point (Figure 3C). There were no significant differences found between the recorded maximum amplitude (Figure 3D) or SNR (Figure 3E) between the rat and mouse recordings over the entire eight weeks or when compared at acute and chronic time intervals.

### Finite Element Analysis of Tissue Strain

**FIGURE 4:**
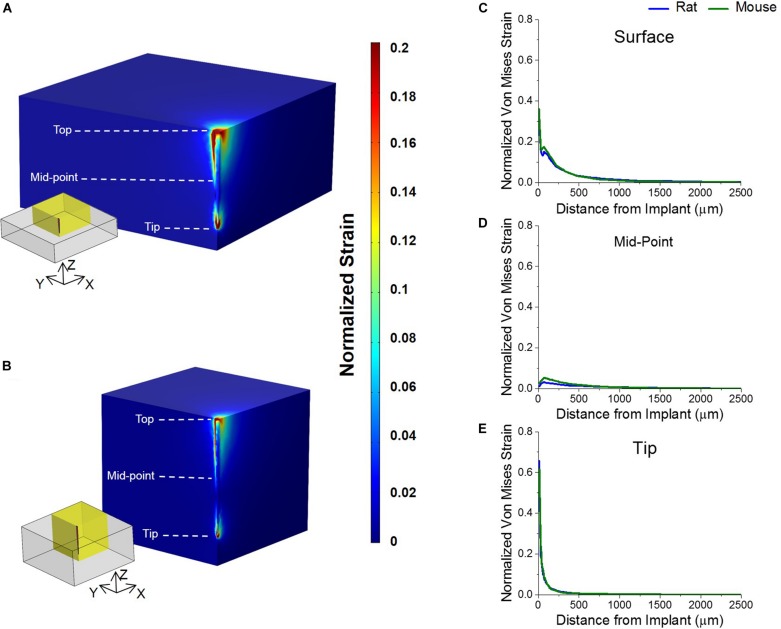
Strain profiles of rat and mouse cortex from implanted silicon probes. Predicted strain profiles induced by a tangential tethering force on silicon probe implanted into **(A)** mouse and **(B)** rat cortex. Normalized strain among a quarter region of each model (as highlighted by yellow insets) were shown are shown. **(C)** The strain of the tissue surrounding the top of the electrode was predicted to be the highest in both animal models. **(D)** The strain around the mid-point of the probe was predicted to be negligible and close to zero in both animal models. **(E)** The strain surrounding the tip of the implanted probe was predicted to be lower than the strain at the top in both animal models. The strain between the two animals was predicted to be similar to each other at all three points analyzed.

Normalized von Mises strain results are as shown in Figure 4. Simulated micromotion around the tethered probe resulted in elevated strains at the tissue surrounding both the top of the brain and tip of the probe (Figures 4A,B). Both animal models predicted to have similar strain profiles to each other around the tissue surrounding the top of the brain, and the mid-point and tip of the probe with a Pearson correlation coefficient of 0.97 (Figures 4C–E). It was found that the highest strain was at the tissue surrounding the top of the brain in both rat and mouse models (Figure 4C).

## Discussion

One of the main challenges of clinical implementation of IME technology is the instability and decline in recording signal (Biran et al., 2005; McConnell et al., 2009; Potter et al., 2012; Kozai et al., 2015b). The rat model is commonly utilized for understanding the decline of recording quality and longevity (Vetter et al., 2004; Nolta et al., 2015; Mahajan et al., 2019; Salatino et al., 2019), as well as for strategies to improve integration at the neural interface to expand electrophysiological outcomes (Jorfi et al., 2015; Kim et al., 2018; Wellman et al., 2018; Won et al., 2018). Recent progress has shown increased use of transgenic, optogenetic and two-photon techniques being employed more frequently in neural interface research (Anikeeva et al., 2011; Kozai et al., 2012, 2014, 2016; Pashaie et al., 2014; Luan et al., 2017; Park et al., 2017; Bedell et al., 2018a, b; Hermann et al., 2018a, b). As advances in neural interface research are made, it is becoming evident that the standardization of rat and mouse models is critical for translation of novel findings. Previous work has shown negligible inflammatory response differences using histological methods between these two animal models (Potter-Baker et al., 2014). However, as the neural interface field is expanding research strategies, it is necessary to further evaluate the differences between these two rodent models. Hence, we conducted a quantitative assessment of the electrophysiological recordings and tissue strain from implanted IMEs in both rat and mouse models.

Electrophysiological recordings were taken twice per week for eight weeks, from awake, freely moving tethered rats and mice. In order to allow for direct comparison between the rat and mouse data, electrophysiological data was analyzed using the same metrics in both groups. Both rodent groups revealed a similar decline in electrode recording, with variability of recordings over time, within each rat and mouse group independently and between the two rodent groups comparatively. Although this observation is not optimal for IME translation, it is not surprising, as recording instability is known to be one of the main challenges with IME use. In fact, similar inconsistent recordings have been observed in other animal models, including cat and primate models (Rousche and Normann, 1998; Chestek et al., 2011; Barrese et al., 2013). It is noteworthy that both rodent models showed similar trends of signal decline and instability over the eight weeks. This indicates that the use of either rodent model will result in similar recording patterns, thus making the two models comparable.

Differences in the percentage of channels recording single unit activity and the number of units recorded per channel between the rat and mouse model were noted within this study. Further analysis revealed these differences were only significant within the first two weeks of the study. In fact, it was observed that the rat model had significantly higher percentage of channels recording single unit activity and the number of units recorded per channel in the first two weeks. However, the two animal models had overlapping trends for the remainder of the study. These observed differences can be explained by a few key differences between the animal models and surgical technique. First, the insertion rates between the two animal models varied slightly, with the rat insertion at 100 μm every 1–2 min and the mouse surgeries at a rate of 10 μm/s every 10–30 s. It has recently been shown that slower insertion rates, 120 μm/min, results in improved recording quality, with the highest and most stable SNR, increased single unit yield, and the highest ratio of inhibitory interneurons at acute time points (1and 45 min) (Fiáth et al., 2019). The insertion rate for the rat model was in line with the optimal insertion rates reported by Fiáth et al. (2019), indicating that the improved recordings observed during the first two weeks after implantation could be in part due to the slower insertion speed. It is thought that the slower insertion speed allows the brain tissue to recover from the necessary compression and stretching forces without breeching the BBB, which ultimately results in more neuronal survival around the implant (Edell et al., 1992; Bjornsson et al., 2006; Fiáth et al., 2019). Accordingly, the slower insertion speed used to implant into the rat may have actually allowed the brain tissue to partially recover during the insertion time, thus resulting in improved recordings during the acute time point. There were no statistically significant differences of percentage of channels recording single unit activity and the number of units recorded per channel between the two rodent models beyond the acute time interval. This further suggests differences at the early time points were not due to inherent differences in the animal models themselves but due to temporary factors associated with implantation techniques. Additionally, it is common practice among some research groups to allow the rodents to recover from surgery for approximately one week prior to performing electrophysiological recordings, which our study did not incorporate, thus negating the acute recording differences observed herein (Nolta et al., 2015; White et al., 2016; Harris et al., 2017). Markedly, the duration of IME implantation for the majority of similar rodent studies is 12-16 weeks, emphasizing the importance of having comparable recordings between the rat and mouse models during the chronic time period (Kozai et al., 2014; Kozai et al., 2015a; Bedell et al., 2018a; Hermann et al., 2018b; Usoro et al., 2019). Furthermore, clinical trials utilizing implanted IMEs remain implanted on a timescale of years, underscoring the value of our chronic findings (Hochberg et al., 2006, 2012; Ajiboye et al., 2017).

The depth that the electrode was inserted varied between the two rodent models. Although the recording contacts on the inserted electrode was proportional to the optimal cortical layers (layers III–V) of the respective rodent model, this variation may have resulted in the acute increase of percentage of channels recording single unit activity and the number of units recorded per channel in the rat model. In a previous study examining the inflammatory response to implanted electrodes between rat and mouse models, it was observed that there were significantly more neurons around the implanted probe in the rat cortex at two weeks compared to the mouse cortex (Potter-Baker et al., 2014). This observation was attributed to the difference in scale between the two animal models, specifically, the thickness of the cortical tissue, size of the cortical layers and the distribution of cells within that space (Defelipe, 2011; Potter-Baker et al., 2014). Furthermore, the variances of the single unit activity between the two animal models can also be attributed to the number of synaptic inputs per neuron in each animal model (DeFelipe et al., 2002; Defelipe, 2011). As follows, this calculated synaptic activity per neuron in various animal models has revealed variances between animal models and cortical layers within the same animal model (DeFelipe et al., 2002; Defelipe, 2011). In fact, there are specific differences in the proportion, length, and density of putative excitatory and inhibitory synapses, which may not affect cortical layers the same, between the rat and mouse (Defelipe, 2011). Although the dissimilarities between the rat and mouse brain scale and synaptic activity could have been a source to the observed differences in electrophysiological recordings, further investigation into this hypothesis is required prior to concluding the observed electrophysiological differences between the two rodent models is due to inherent anatomy and physiology of the animals. The fact that there were no observed significant differences in the chronic recording metrics of percentage of channels recording single unit activity, number of units recorded per channel, SNR and amplitude between the two rodent models would suggest the cyto-architecture differences between models may only have a limited effect on recording differences. The greater increase in recorded noise with the mouse model compared to the rat model may have been confounded by differences in environmental noise between the two models since the mice recordings were performed in a class II sterile hood and the rats were recorded on a non-enclosed surgical table. Hence, the overall electrophysiological results comparing the rat and mouse model show negligible differences between the two models and encourage future investigations to utilize either or both animal models.

In order to predict the micromotion induced strains surrounding the implanted probe, a finite element model simulating a 20 μm displacement to the top of the probe was utilized. The differences in electrode implantation depth, thickness of the cortex, and marginal variances in electrode geometry were all taken into consideration in the models. Interestingly, there were negligible differences between the predicted strain for the two rodent models. These results indicate that the strain induced onto the tissue from respiratory and vasculature micromotion is negligible between the rat and mouse. Both models displayed similar strain profiles with the highest being at the top of the brain (i.e., top of the probe), similar to our previous findings (Nguyen et al., 2014). Conversely, a variable that was not included in the model, but differed between the two rodent models is the reflection of the dura. During the rat surgeries, the dura is reflected to allow for ease of electrode insertion due to the stiffness of the dura. However, in the mouse, the dura is much thinner and removal is not necessary for electrode implantation. The influence reflection of the dura has on tissue strain, micromotion, and downstream inflammatory response is not fully understood, but likely has some effect initially before the dura regrows over time. In fact, Gilletti and Muthuswamy (2006) showed the presence of the dura exhibited a significantly lower respiratory displacement of rat brain tissue, compared to animals without a dura. However, the presence of the dura had no effect on the vasculature displacement (Gilletti and Muthuswamy, 2006). Our model did not take into account the different displacement values between the rat with a reflected dura and the mouse with an intact dura. Our model used total displacement, combining the effects of both vasculature and respiration, since the vasculature displacement has not been shown to be influenced by dura reflection; we kept the displacement value constant for both models. In future work, experimental measurements of displacement from both rat and mouse under conditions of reflected or intact dura will be conducted and the experimental data will be used as model inputs to define the brain micromotion. Future finite element models can incorporate multi-physics models, in which the effect of tissue strain from physiological micromotion and/or material stiffness can be correlated to the electrophysiological recordings. In fact, Lempka et al. (2011) utilized multi-physics computational modeling to investigate potential factors, such as electrode geometry and noise sources, to identify their effect on recording quality. The effects on electrophysiology from pathophysiology of the brain, such as the formed glial scar, can also be assessed through multi-physics models. For instance, computational modeling from Malaga et al. (2015) discovered that the glial scar did not contribute to the reduction of signal amplitude, instead it was suggested that the displacement of neurons from the electrode was the main contributor to the size of the amplitude (Malaga et al., 2015). The work by Malaga et al. (2015) in combination with the total findings from Potter-Baker et al. (2014) demonstrating significantly more neurons around the implanted probe in the rat cortex at two weeks and the current findings herein signifying improved recording quality from the rat during the first two weeks, alludes that the improved recordings in the rat model are possibly due to less neuronal displacement after electrode implantation. Future multiple physics computational modeling, as well as experimental approaches, can further assess the acute neuronal displacement and coinciding electrophysiological recordings between animal models, while also taking into consideration the effects from reflection of the dura, tissue strain, glial scar, among many other variables.

## Conclusion

Collectively the electrophysiological and predictive tissue strain data reveal there are negligible differences between the rat and mouse models. The reported increase of percentage of channels recording single unit activity and the number of units recorded per channel between the rat and mouse model could be attributed to the differences in electrode insertion rate. However, this variance observed may also be due to inherent differences in animal scale and physiology, but this requires further investigation. Finally, the finite element model revealed negligible differences between the two rodent models tissue strain from implanted electrodes. Although, the reflection of the dura in the rat model was not directly compared to the intact dura in the mouse model, dural regrowth after implantation should make the two models representative of strain at chronic time points. The finite element model presented herein, although preliminary, can set the foundation for future multiple physics components to be incorporated into.

## Data Availability Statement

The datasets generated for this study are available on request to the corresponding author.

## Ethics StaTement

The animal study was reviewed and approved by the Institutional Animal Care and Use Committee (IACUC) at the Louis Stokes Cleveland Veterans Affairs Medical Center (LSCDVAMC) and Case Western Reserve University (CWRU).

## Author Contributions

EE and JC contributed substantially to the conception and design of the work, analysis, and interpretation of data for the work, drafting the manuscript for important intellectual content, approved the final version to be published and agreed to be accountable for all aspects of the work. JH and HB contributed to the set-up, performance and analysis of the mouse electrophysiology data, as well as contributed to the conception, design and interpretation of the work. JS and SM each drafted sections of the manuscript. SM, LC, JH, and MS contributed to the design, implantation, and interpretation of the finite element strain models. EE, CS, SMM, JR, KC, HF, YK, and DT helped with the set-up, acquisition and analysis of the rat electrophysiology data. All authors approved the final version to be published and agreed to be accountable for all aspects of the work.

## Conflict of Interest

The authors declare that the research was conducted in the absence of any commercial or financial relationships that could be construed as a potential conflict of interest.

## References

[B1] Ajiboye A. B. Bolu Ajiboye A. Willett F. R. Young D. R. Memberg W. D. Murphy B. A. (2017). Restoration of reaching and grasping movements through brain-controlled muscle stimulation in a person with tetraplegia: a proof-of-concept demonstration. *Lancet* 389 1821–1830. 10.1016/S0140-6736(17)30601-3 28363483 PMC5516547

[B2] Anikeeva P. Andalman A. S. Witten I. Warden M. Goshen I. Grosenick L. (2011). Optetrode: a multichannel readout for optogenetic control in freely moving mice. *Nat. Neurosci.* 15 163–170. 10.1038/nn.2992 22138641 PMC4164695

[B3] Barrese J. C. Aceros J. Donoghue J. P. (2016). Scanning electron microscopy of chronically implanted intracortical microelectrode arrays in non-human primates. *J. Neural Eng.* 13:026003. 10.1088/1741-2560/13/2/026003 26824680 PMC4854331

[B4] Barrese J. C. Rao N. Paroo K. Triebwasser C. Vargas-Irwin C. Franquemont L. (2013). Failure mode analysis of silicon-based intracortical microelectrode arrays in non-human primates. *J. Neural Eng.* 10:066014. 10.1088/1741-2560/10/6/066014 24216311 PMC4868924

[B5] Barre-Sinoussi F. Montagutelli X. (2015). Animal models are essential to biological research: issues and perspectives. *Future Sci.* 1:Fso63. 10.4155/fso.15.63 28031915 PMC5137861

[B6] Bedell H. W. Hermann J. K. Ravikumar M. Lin S. Rein A. Li X. (2018a). Targeting CD14 on blood derived cells improves intracortical microelectrode performance. *Biomaterials* 163 163–173. 10.1016/j.biomaterials.2018.02.014 29471127 PMC5841759

[B7] Bedell H. W. Song S. Li X. Molinich E. Lin S. Stiller A. (2018b). Understanding the effects of both CD14-mediated innate immunity and device/tissue mechanical mismatch in the neuroinflammatory response to intracortical microelectrodes. *Front. Neurosci.* 12:772. 10.3389/fnins.2018.00772 30429766 PMC6220032

[B8] Bedell H. W. Schaub N. Capadona J. R. Ereifej E. S. (2019). Differential expression of genes involved in the acute innate immune response to intracortical microelectrodes. *Acta Biomater.* 102 205–219. 10.1016/j.actbio.2019.11.017 31733330 PMC6944063

[B9] Bennett C. Mohammed F. Álvarez-Ciara A. Nguyen M. A. Dietrich W. D. Rajguru S. M. (2019). Neuroinflammation, oxidative stress, and blood-brain barrier (BBB) disruption in acute Utah electrode array implants and the effect of deferoxamine as an iron chelator on acute foreign body response. *Biomaterials* 188 144–159. 10.1016/j.biomaterials.2018.09.040 30343257 PMC6300159

[B10] Bennett C. Samikkannu M. Mohammed F. Dietrich W. D. Rajguru S. M. Prasad A. (2018). Blood brain barrier (BBB)-disruption in intracortical silicon microelectrode implants. *Biomaterials* 164 1–10. 10.1016/j.biomaterials.2018.02.036 29477707 PMC5895107

[B11] Biran R. Martin D. C. Tresco P. A. (2005). Neuronal cell loss accompanies the brain tissue response to chronically implanted silicon microelectrode arrays. *Exp. Neurol.* 195 115–126. 10.1016/j.expneurol.2005.04.020 16045910

[B12] Bjornsson C. Oh S. J. Al-Kofahi Y. Lim Y. Smith K. Turner J. (2006). Effects of insertion conditions on tissue strain and vascular damage during neuroprosthetic device insertion. *J. Neural Eng.* 3:196. 10.1088/1741-2560/3/3/002 16921203

[B13] Buzsáki G. (2004). Large-scale recording of neuronal ensembles. *Nat. Neurosci.* 7:446. 15114356 10.1038/nn1233

[B14] Chestek C. A. Gilja V. Nuyujukian P. Foster J. D. Fan J. M. Kaufman M. T. (2011). Long-term stability of neural prosthetic control signals from silicon cortical arrays in rhesus macaque motor cortex. *J. Neural Eng.* 8:045005. 10.1088/1741-2560/8/4/045005 21775782 PMC3644617

[B15] Debnath S. Prins N. W. Pohlmeyer E. Mylavarapu R. Geng S. Sanchez J. C. (2018). Long-term stability of neural signals from microwire arrays implanted in common marmoset motor cortex and striatum. *Biomed. Phys. Eng. Exp.* 4:055025. 10.1088/2057-1976/aada67 31011432 PMC6474681

[B16] Defelipe J. (2011). The evolution of the brain, the human nature of cortical circuits, and intellectual creativity. *Front. Neuroanat.* 5:29. 10.3389/fnana.2011.00029 21647212 PMC3098448

[B17] DeFelipe J. Alonso-Nanclares L. Arellano J. I. (2002). Microstructure of the neocortex: comparative aspects. *J. Neurocytol.* 31 299–316. 10.1023/a:1024130211265 12815249

[B18] Edell D. J. Toi V. Mcneil V. M. Clark L. (1992). Factors influencing the biocompatibility of insertable silicon microshafts in cerebral cortex. *IEEE Trans. Biomed. Eng.* 39 635–643. 10.1109/10.141202 1601445

[B19] Ereifej E. S. Rial G. M. Hermann J. K. Smith C. S. Meade S. M. Rayyan J. M. (2018). Implantation of neural probes in the brain elicits oxidative stress. *Front. Bioeng. Biotechnol.* 6:9. 10.3389/fbioe.2018.00009 29487848 PMC5816578

[B20] Ereifej E. S. Smith C. S. Meade S. M. Chen K. Feng H. Capadona J. R. (2017). The neuroinflammatory response to nanopatterning parallel grooves into the surface structure of intracortical microelectrodes. *Adv. Funct. Mater.* 28:1704420.

[B21] Fiáth R. Márton A. L. Mátyás F. Pinke D. Márton G. Tóth K. (2019). Slow insertion of silicon probes improves the quality of acute neuronal recordings. *Sci. Rep.* 9:111. 10.1038/s41598-018-36816-z 30643182 PMC6331571

[B22] Giannobile W. V. Finkelman R. D. Lynch S. E. (1994). Comparison of canine and non-human primate animal models for periodontal regenerative therapy: results following a single administration of PDGF/IGF-I. *J. Periodontol.* 65 1158–1168. 10.1902/jop.1994.65.12.1158 7877089

[B23] Gilja V. Pandarinath C. Blabe C. H. Nuyujukian P. Simeral J. D. Sarma A. A. (2015). Clinical translation of a high-performance neural prosthesis. *Nat. Med.* 21:1142. 10.1038/nm.3953 26413781 PMC4805425

[B24] Gilletti A. Muthuswamy J. (2006). Brain micromotion around implants in the rodent somatosensory cortex. *J. Neural Eng.* 3 189–195. 10.1088/1741-2560/3/3/001 16921202

[B25] Goss-Varley M. Dona K. R. Mcmahon J. A. Shoffstall A. J. Ereifej E. S. Lindner S. C. (2017). Microelectrode implantation in motor cortex causes fine motor deficit: implications on potential considerations to brain computer interfacing and human augmentation. *Sci. Rep.* 7:15254. 10.1038/s41598-017-15623-y 29127346 PMC5681545

[B26] Goss-Varley M. Shoffstall A. J. Dona K. R. Mcmahon J. A. Lindner S. C. Ereifej E. S. (2018). Rodent behavioral testing to assess functional deficits caused by microelectrode implantation in the rat motor cortex. *J. Vis. Exp.* 138:57829. 10.3791/57829 30176008 PMC6128113

[B27] Harris A. Z. Golder D. Likhtik E. (2017). Multisite electrophysiology recordings in mice to study cross-regional communication during anxiety. *Curr. Protoc. Neurosci.* 80 8.40.1–48.40.21. 10.1002/cpns.32 28678397 PMC5783183

[B28] Harris J. P. Capadona J. R. Miller R. H. Healy B. C. Shanmuganathan K. Rowan S. J. (2011). Mechanically adaptive intracortical implants improve the proximity of neuronal cell bodies. *J. Neural Eng.* 8:066011. 10.1088/1741-2560/8/6/066011 22049097 PMC3386315

[B29] He W. Mcconnell G. C. Schneider T. M. Bellamkonda R. V. (2007). A novel anti-inflammatory surface for neural electrodes. *Adv. Mater.* 19 3529–3533.

[B30] Hermann J. K. Lin S. Soffer A. Wong C. Srivastava V. Chang J. (2018a). The role of toll-like receptor 2 and 4 innate immunity pathways in intracortical microelectrode-induced neuroinflammation. *Front. Bioeng. Biotechnol.* 6:113. 10.3389/fbioe.2018.00113 30159311 PMC6104445

[B31] Hermann J. K. Ravikumar M. Shoffstall A. J. Ereifej E. S. Kovach K. M. Chang J. (2018b). Inhibition of the cluster of differentiation 14 innate immunity pathway with IAXO-101 improves chronic microelectrode performance. *J. Neural Eng.* 15:025002. 10.1088/1741-2552/aaa03e 29219114 PMC5818286

[B32] Hochberg L. R. Bacher D. Jarosiewicz B. Masse N. Y. Simeral J. D. Vogel J. (2012). Reach and grasp by people with tetraplegia using a neurally controlled robotic arm. *Nature* 485 372–375. 10.1038/nature11076 22596161 PMC3640850

[B33] Hochberg L. R. Serruya M. D. Friehs G. M. Mukand J. A. Saleh M. Caplan A. H. (2006). Neuronal ensemble control of prosthetic devices by a human with tetraplegia. *Nature* 442 164–171. 10.1038/nature04970 16838014

[B34] Islam M. S. Koya D. Portha B. (2013). Animal models of diabetes and its associated complications. *J. Diabetes Res.* 2013:593204. 10.1155/2013/593204 24288690 PMC3830851

[B35] Jorfi M. Skousen J. L. Weder C. Capadona J. R. (2015). Progress towards biocompatible intracortical microelectrodes for neural interfacing applications. *J. Neural Eng.* 12:011001. 10.1088/1741-2560/12/1/011001 25460808 PMC4428498

[B36] Kim Y. Meade S. M. Chen K. Feng H. Rayyan J. Hess-Dunning A. (2018). Nano-architectural approaches for improved intracortical interface technologies. *Front. Neurosci.* 12:456. 10.3389/fnins.2018.00456 30065623 PMC6056633

[B37] Kipke D. R. Shain W. Buzsáki G. Fetz E. Henderson J. M. Hetke J. F. (2008). Advanced neurotechnologies for chronic neural interfaces: new horizons and clinical opportunities. *J. Neurosci.* 28 11830–11838. 10.1523/JNEUROSCI.3879-08.2008 19005048 PMC3844837

[B38] Kozai T. D. Du Z. Gugel Z. V. Smith M. A. Chase S. M. Bodily L. M. (2015a). Comprehensive chronic laminar single-unit, multi-unit, and local field potential recording performance with planar single shank electrode arrays. *J. Neurosci. Methods* 242 15–40. 10.1016/j.jneumeth.2014.12.010 25542351 PMC4432916

[B39] Kozai T. D. Y. Jaquins-Gerstl A. S. Vazquez A. L. Michael A. C. Cui X. T. (2015b). Brain tissue responses to neural implants impact signal sensitivity and intervention strategies. *ACS Chem. Neurosci.* 6 48–67. 10.1021/cn500256e 25546652 PMC4304489

[B40] Kozai T. D. Eles J. R. Vazquez A. L. Cui X. T. (2016). Two-photon imaging of chronically implanted neural electrodes: sealing methods and new insights. *J. Neurosci. Methods* 258 46–55. 10.1016/j.jneumeth.2015.10.007 26526459 PMC4771525

[B41] Kozai T. D. Li X. Bodily L. M. Caparosa E. M. Zenonos G. A. Carlisle D. L. (2014). Effects of caspase-1 knockout on chronic neural recording quality and longevity: insight into cellular and molecular mechanisms of the reactive tissue response. *Biomaterials* 35 9620–9634. 10.1016/j.biomaterials.2014.08.006 25176060 PMC4174599

[B42] Kozai T. D. Vazquez A. L. Weaver C. L. Kim S. G. Cui X. T. (2012). In vivo two-photon microscopy reveals immediate microglial reaction to implantation of microelectrode through extension of processes. *J. Neural Eng.* 9:066001. 10.1088/1741-2560/9/6/066001 23075490 PMC3511663

[B43] Lee H. Bellamkonda R. V. Sun W. Levenston M. E. (2005). Biomechanical analysis of silicon microelectrode-induced strain in the brain. *J. Neural Eng.* 2 81–89. 10.1088/1741-2560/2/4/003 16317231

[B44] Lempka S. F. Johnson M. D. Moffitt M. A. Otto K. J. Kipke D. R. Mcintyre C. C. (2011). Theoretical analysis of intracortical microelectrode recordings. *J. Neural Eng.* 8:045006. 10.1088/1741-2560/8/4/045006 21775783 PMC3196618

[B45] Luan L. Wei X. Zhao Z. Siegel J. J. Potnis O. Tuppen C. A. (2017). Ultraflexible nanoelectronic probes form reliable, glial scar–free neural integration. *Sci. Adv.* 3:e1601966. 10.1126/sciadv.1601966 28246640 PMC5310823

[B46] MacManus D. B. Pierrat B. Murphy J. G. Gilchrist M. D. (2017). Region and species dependent mechanical properties of adolescent and young adult brain tissue. *Sci. Rep.* 7 1–12. 10.1038/s41598-018-28932-7 29061984 PMC5653834

[B47] Mahajan S. Sharkins J. A. Hunter A. H. Avishai A. Ereifej E. S. (2019). Focused ion beam lithography to etch nano-architectures into microelectrodes. *J. Vis. Exp.* 155:e60004. 10.3791/60004 32009634 PMC8457512

[B48] Malaga K. A. Schroeder K. E. Patel P. R. Irwin Z. T. Thompson D. E. Bentley J. N. (2015). Data-driven model comparing the effects of glial scarring and interface interactions on chronic neural recordings in non-human primates. *J. Neural Eng.* 13:016010. 10.1088/1741-2560/13/1/016010 26655972

[B49] McConnell G. C. Rees H. D. Levey A. I. Gutekunst C.-A. Gross R. E. Bellamkonda R. V. (2009). Implanted neural electrodes cause chronic, local inflammation that is correlated with local neurodegeneration. *J. Neural Eng.* 6:056003. 10.1088/1741-2560/6/5/056003 19700815

[B50] Michelson N. J. Kozai T. D. (2018). Isoflurane and ketamine differentially influence spontaneous and evoked laminar electrophysiology in mouse V1. *J. Neurophysiol.* 120 2232–2245. 10.1152/jn.00299.2018 30067128 PMC6295540

[B51] Muthuswamy J. Gilletti A. Jain T. Okandan M. (2003). “Microactuated neural probes to compensate for brain micromotion,” in *Proceedings of the 25th Annual International Conference of the IEEE Engineering in Medicine and Biology Society (IEEE Cat. No. 03CH37439)*, (Cancun: IEEE), 1941–1943.

[B52] Newman E. Turner A. Wark J. (1995). The potential of sheep for the study of osteopenia: current status and comparison with other animal models. *Bone* 16 S277–S284. 10.1016/8756-3282(95)00026-a 7626315

[B53] Nguyen J. K. Jorfi M. Buchanan K. L. Park D. J. Foster E. J. Tyler D. J. (2016). Influence of resveratrol release on the tissue response to mechanically adaptive cortical implants. *Acta Biomater.* 29 81–93. 10.1016/j.actbio.2015.11.001 26553391 PMC4727752

[B54] Nguyen J. K. Park D. J. Skousen J. L. Hess-Dunning A. Tyler D. J. Rowan S. J. (2014). Mechanically-compliant intracortical implants reduce the neuroinflammatory response. *J. Neural Eng.* 11:056014. 10.1088/1741-2560/11/5/056014 25125443 PMC4175058

[B55] Nolta N. F. Christensen M. B. Crane P. D. Skousen J. L. Tresco P. A. (2015). BBB leakage, astrogliosis, and tissue loss correlate with silicon microelectrode array recording performance. *Biomaterials* 53 753–762. 10.1016/j.biomaterials.2015.02.081 25890770

[B56] Park S. Guo Y. Jia X. Choe H. K. Grena B. Kang J. (2017). One-step optogenetics with multifunctional flexible polymer fibers. *Nat. Neurosci.* 20 612–619. 10.1038/nn.4510 28218915 PMC5374019

[B57] Pashaie R. Anikeeva P. Lee J. H. Prakash R. Yizhar O. Prigge M. (2014). Optogenetic brain interfaces. *IEEE Rev. Biomed. Eng.* 7 3–30. 10.1109/RBME.2013.2294796 24802525

[B58] Potter K. A. Buck A. C. Self W. K. Callanan M. E. Sunil S. Capadona J. R. (2013). The effect of resveratrol on neurodegeneration and blood brain barrier stability surrounding intracortical microelectrodes. *Biomaterials* 34 7001–7015. 10.1016/j.biomaterials.2013.05.035 23791503

[B59] Potter K. A. Buck A. C. Self W. K. Capadona J. R. (2012). Stab injury and device implantation within the brain results in inversely multiphasic neuroinflammatory and neurodegenerative responses. *J. Neural Eng.* 9:046020. 10.1088/1741-2560/9/4/046020 22832283

[B60] Potter-Baker K. A. Ravikumar M. Burke A. A. Meador W. D. Householder K. T. Buck A. C. (2014). A comparison of neuroinflammation to implanted microelectrodes in rat and mouse models. *Biomaterials* 35 5637–5646. 10.1016/j.biomaterials.2014.03.076 24755527 PMC4071936

[B61] Prasad A. Xue Q.-S. Dieme R. Sankar V. Mayrand R. C. Nishida T. (2014). Abiotic-biotic characterization of Pt/Ir microelectrode arrays in chronic implants. *Front. Neuroeng.* 7:2. 10.3389/fneng.2014.00002 24550823 PMC3912984

[B62] Prodanov D. Delbeke J. (2016). Mechanical and biological interactions of implants with the brain and their impact on implant design. *Front. Neurosci.* 10:11. 10.3389/fnins.2016.00011 26903786 PMC4746296

[B63] Quiroga R. Q. Nadasdy Z. Ben-Shaul Y. (2004). Unsupervised spike detection and sorting with wavelets and superparamagnetic clustering. *Neural Comput.* 16 1661–1687. 10.1162/089976604774201631 15228749

[B64] Ravikumar M. Hageman D. J. Tomaszewski W. H. Chandra G. M. Skousen J. L. Capadona J. R. (2014a). The effect of residual endotoxin contamination on the neuroinflammatory response to sterilized intracortical microelectrodes. *J. Mater. Chem. B* 2 2517–2529. 24778808 10.1039/C3TB21453B PMC4000032

[B65] Ravikumar M. Sunil S. Black J. Barkauskas D. S. Haung A. Y. Miller R. H. (2014b). The roles of blood-derived macrophages and resident microglia in the neuroinflammatory response to implanted intracortical microelectrodes. *Biomaterials* 35 8049–8064. 10.1016/j.biomaterials.2014.05.084 24973296 PMC4169074

[B66] Reichert W. M. (2007). *Indwelling Neural Implants: Strategies for Contending With the in vivo Environment.* Boca Raton, FL: CRC Press. 21204399

[B67] Renshaw B. Forbes A. Morison B. R. (1940). Activity of isocortex and hippocampus: electrical studies with micro-electrodes. *J. Neurophysiol.* 3 74–105.

[B68] Rousche P. J. Normann R. A. (1998). Chronic recording capability of the utah intracortical electrode array in cat sensory cortex. *J. Neurosci. Methods* 82 1–15. 10223510 10.1016/s0165-0270(98)00031-4

[B69] Salatino J. W. Kale A. P. Purcell E. K. (2019). Alterations in ion channel expression surrounding implanted microelectrode arrays in the brain. *bioRxiv* [Preprint] 10.1101/518811

[B70] Saxena T. Karumbaiah L. Gaupp E. A. Patkar R. Patil K. Betancur M. (2013). The impact of chronic blood-brain barrier breach on intracortical electrode function. *Biomaterials* 34 4703–4713. 10.1016/j.biomaterials.2013.03.007 23562053

[B71] Schroeder K. E. Chestek C. A. (2016). Intracortical brain-machine interfaces advance sensorimotor neuroscience. *Front. Neurosci.* 10:291. 10.3389/fnins.2016.00291 27445663 PMC4923184

[B72] Schwartz A. B. (2004). Cortical neural prosthetics. *Annu. Rev. Neurosci.* 27 487–507. 15217341 10.1146/annurev.neuro.27.070203.144233

[B73] Schwartz A. B. Cui X. T. Weber D. J. Moran D. W. (2006). Brain-controlled interfaces: movement restoration with neural prosthetics. *Neuron* 52 205–220. 17015237 10.1016/j.neuron.2006.09.019

[B74] Shanks N. Greek R. Greek J. (2009). Are animal models predictive for humans? *Philos. Ethics Human. Med.* 4:2. 10.1186/1747-5341-4-2 19146696 PMC2642860

[B75] Skousen J. L. Bridge M. J. Tresco P. A. (2015). A strategy to passively reduce neuroinflammation surrounding devices implanted chronically in brain tissue by manipulating device surface permeability. *Biomaterials* 36 33–43. 10.1016/j.biomaterials.2014.08.039 25310936

[B76] Sridharan A. Rajan S. D. Muthuswamy J. (2013). Long-term changes in the material properties of brain tissue at the implant–tissue interface. *J. Neural Eng.* 10:066001. 10.1088/1741-2560/10/6/066001 24099854 PMC3888957

[B77] Subbaroyan J. Martin D. C. Kipke D. R. (2005). A finite-element model of the mechanical effects of implantable microelectrodes in the cerebral cortex. *J. Neural Eng.* 2 103–113. 16317234 10.1088/1741-2560/2/4/006

[B78] Usoro J. O. Shih E. Black B. J. Rihani R. T. Abbott J. Chakraborty B. (2019). Chronic stability of local field potentials from standard and modified Blackrock microelectrode arrays implanted in the rat motor cortex. *Biomed. Phys. Eng. Exp.* 5:065017.

[B79] Vetter R. J. Williams J. C. Nunamaker E. A. Kipke D. R. (2004). Chronic neural recording using silicon-substrate microelectrode arrays implanted in cerebral cortex. *IEEE Trans. Biomed. Eng.* 51 896–904. 15188856 10.1109/TBME.2004.826680

[B80] Wellman S. M. Eles J. R. Ludwig K. A. Seymour J. P. Michelson N. J. Mcfadden W. E. (2018). A materials roadmap to functional neural interface design. *Adv. Funct. Mater.* 28:1701269. 10.1002/adfm.201701269 29805350 PMC5963731

[B81] White J. J. Lin T. Brown A. M. Arancillo M. Lackey E. P. Stay T. L. (2016). An optimized surgical approach for obtaining stable extracellular single-unit recordings from the cerebellum of head-fixed behaving mice. *J. Neurosci. Methods* 262 21–31. 10.1016/j.jneumeth.2016.01.010 26777474 PMC4778558

[B82] Winslow B. D. Tresco P. A. (2010). Quantitative analysis of the tissue response to chronically implanted microwire electrodes in rat cortex. *Biomaterials* 31 1558–1567. 10.1016/j.biomaterials.2009.11.049 19963267

[B83] Wise K. D. (2005). Silicon microsystems for neuroscience and neural prostheses. *IEEE Eng. Med. Biol. Mag.* 24 22–29. 10.1109/memb.2005.1511497 16248114

[B84] Wise K. D. Angell J. B. (1975). A low-capacitance multielectrode probe for use in extracellular neurophysiology. *IEEE Trans. Biomed. Eng.* 22 212–219. 1116854 10.1109/tbme.1975.324562

[B85] Won S. M. Song E. Zhao J. Li J. Rivnay J. Rogers J. A. (2018). Recent advances in materials, devices, and systems for neural interfaces. *Adv. Mater.* 30:1800534. 10.1002/adma.201800534 29855089

[B86] Working P. K. (1988). Male reproductive toxicology: comparison of the human to animal models. *Environ. Health Perspect.* 77 37–44. 3289906 10.1289/ehp.887737 PMC1474524

[B87] Xindong L. Mccreery D. B. Bullara L. A. Agnew W. F. (2006). Evaluation of the stability of intracortical microelectrode arrays. *IEEE Trans. Neural. Syst. Rehabil. Eng.* 14 91–100. 16562636 10.1109/TNSRE.2006.870495

[B88] Xindong L. Mccreery D. B. Carter R. R. Bullara L. A. Yuen T. G. H. Agnew W. F. (1999). Stability of the interface between neural tissue and chronically implanted intracortical microelectrodes. *IEEE Trans. Rehabil. Eng.* 7 315–326. 10498377 10.1109/86.788468

[B89] Yan G. Zhang G. Fang X. Zhang Y. Li C. Ling F. (2011). Genome sequencing and comparison of two nonhuman primate animal models, the cynomolgus and Chinese rhesus macaques. *Nat. Biotechnol.* 29 1019–1023. 10.1038/nbt.1992 22002653

[B90] Zhang W. Li Z. Gilles M. Wu D. (2014). Mechanical simulation of neural electrode-brain tissue interface under various micromotion conditions. *J. Med. Biol. Eng.* 34 386–392.

[B91] Zhong Y. Bellamkonda R. V. (2007). Dexamethasone-coated neural probes elicit attenuated inflammatory response and neuronal loss compared to uncoated neural probes. *Brain Res.* 1148 15–27. 17376408 10.1016/j.brainres.2007.02.024 PMC1950487

